# (2-Chloro-8-meth­oxy­quinolin-3-yl)methanol monohydrate

**DOI:** 10.1107/S1600536810020489

**Published:** 2010-06-05

**Authors:** S. Mohana Roopan, F. Nawaz Khan, A. Sudheer Kumar, Venkatesha R. Hathwar, Mehmet Akkurt

**Affiliations:** a Organic and Medicinal Chemistry Research Laboratory, Organic Chemistry Division, School of Advanced Sciences, VIT University, Vellore 632 014, Tamil Nadu, India; b Solid State and Structural Chemistry Unit, Indian Institute of Science, Bangalore 560 012, Karnataka, India; c Department of Physics, Faculty of Arts and Sciences, Erciyes University, 38039 Kayseri, Turkey

## Abstract

In the title compound, C_11_H_10_ClNO_2_·H_2_O, the organic mol­ecule is roughly planar (r.m.s. deviation = 0.074 Å). In the crystal structure, molecues are linked by O—H⋯O and O—H⋯N hydrogen bonds and weak C—H⋯π and π–π inter­actions [centroid–centroid distance = 3.578 (3) Å] consolidate the packing. A short Cl⋯O contact [3.147 (3) Å] is also observed.

## Related literature

For further information on the starting material, see: Subashini *et al.* (2009 ▶). For general background to the title compound, see: Roopan *et al.* (2009 ▶). For related structures, see: Khan *et al.* (2010*a* ▶,*b* ▶). For bond-length data, see: Allen *et al.* (1987 ▶).
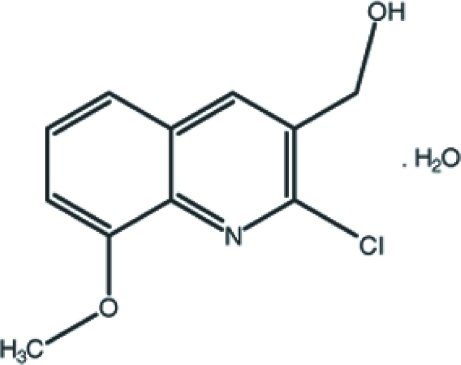


## Experimental

### 

#### Crystal data

C_11_H_10_ClNO_2_·H_2_O
*M* *_r_* = 241.67
Monoclinic, 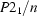

*a* = 9.161 (5) Å
*b* = 14.246 (5) Å
*c* = 9.464 (5) Å
β = 116.819 (5)°
*V* = 1102.3 (9) Å^3^
*Z* = 4
Mo *K*α radiation
μ = 0.34 mm^−1^
*T* = 290 K
0.31 × 0.21 × 0.10 mm

#### Data collection

Oxford Xcalibur Eos (Nova) CCD detector diffractometer
Absorption correction: multi-scan (*CrysAlis PRO RED*; Oxford Diffraction, 2009 ▶) *T*_min_ = 0.903, *T*_max_ = 0.967
8360 measured reflections
2044 independent reflections
1212 reflections with *I* > 2σ(*I*)
*R*_int_ = 0.100

#### Refinement

*R*[*F* ^2^ > 2σ(*F* ^2^)] = 0.054
*wR*(*F* ^2^) = 0.134
*S* = 0.90
2044 reflections
153 parameters
H atoms treated by a mixture of independent and constrained refinement
Δρ_max_ = 0.40 e Å^−3^
Δρ_min_ = −0.26 e Å^−3^

### 

Data collection: *CrysAlis PRO CCD* (Oxford Diffraction, 2009 ▶); cell refinement: *CrysAlis PRO CCD*; data reduction: *CrysAlis PRO RED* (Oxford Diffraction, 2009 ▶); program(s) used to solve structure: *SHELXS97* (Sheldrick, 2008 ▶); program(s) used to refine structure: *SHELXL97* (Sheldrick, 2008 ▶); molecular graphics: *ORTEP-3* (Farrugia, 1997 ▶); software used to prepare material for publication: *WinGX* (Farrugia, 1999 ▶).

## Supplementary Material

Crystal structure: contains datablocks global, I. DOI: 10.1107/S1600536810020489/hb5469sup1.cif

Structure factors: contains datablocks I. DOI: 10.1107/S1600536810020489/hb5469Isup2.hkl

Additional supplementary materials:  crystallographic information; 3D view; checkCIF report

## Figures and Tables

**Table 1:** Hydrogen-bond geometry (Å, °) *Cg*1 is the centroid of the N1/C1–C3/C8/C9 ring.

*D*—H⋯*A*	*D*—H	H⋯*A*	*D*⋯*A*	*D*—H⋯*A*
O1—H1*O*⋯O3^i^	0.82	1.90	2.705 (4)	165
O3—H1*W*⋯N1^ii^	0.85 (5)	2.17 (5)	2.988 (4)	163 (4)
O3—H2*W*⋯O1^iii^	0.83 (4)	2.02 (4)	2.836 (4)	171 (5)
C10—H10*B*⋯*Cg*1^i^	0.97	2.93	3.738 (5)	142

Symmetry codes: (i) 
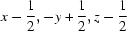
; (ii) 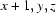; (iii) 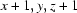.

**Fig. 1.**
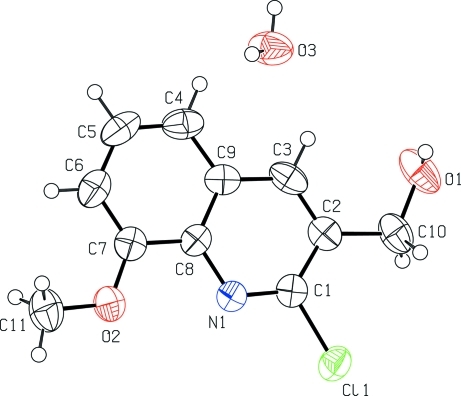
The molecular structure of (I), showing 50% probability displacement ellipsoids.

**Fig. 2.**
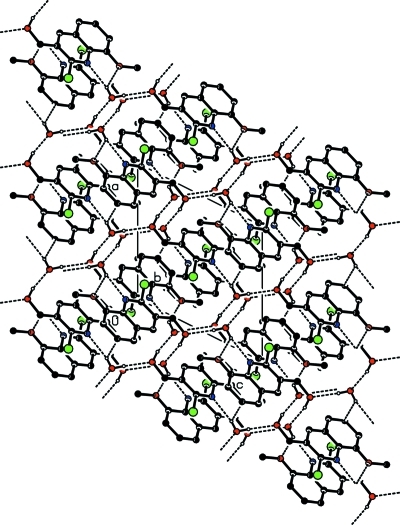
Molecular packing of (I) with hydrogen bonding shown as dashed lines. The H atoms not involved in hydrogen bonds have been omitted for clarity.

## supplementary crystallographic information

### Comment

The importance and general background of the title compound is given in our earler paper (Roopan *et al.*, 2009).

In the main molecule of the title compound (I), (Fig. 1), all the non-H atoms are roughly coplanar (r.m.s. deviation = 0.074 Å). The bond lengths and angles are comparable to the similar structures 2-chloro-3-hydroxymethyl-7,8-dimethylquinoline and 2-chloro-3-hydroxymethyl-6-methoxyquinoline (Khan *et al.*, 2010*a*,b), and also those in literature (Allen *et al.*, 1987).

The crystal structure is stabilized by intermolecular O—H···O and O—H···N interactions between the symmetry-related molecues (Table 1, Fig. 2). Adjacent molecules are stacked along the *b* axis through weak C—H···π interactions (Table 1) and π-π interactions [*Cg*1···*Cg*2(-*x*, 1 - *y*, -*z*) = 3.578 (3) Å, where *Cg*1 and *Cg*2 are centroids of the N1/C1–C3/C8/C9 and C4–C9 rings, respectively]. In addition a short Cl1.. O2 contact of 3.15 Å is also observed.

### Experimental

2-Chloro-8-methoxyquinoline-3-carbaldehyde (222 mg, 1 mmol), sodium borohydride (38 mg, 1 mmol) and catalytic amount of montmorillonite K-10 were taken in an open vessel and the resulting mixture was irradiated at 500 W for 5 min. Ethylacetate was poured into the reaction mixture and filtered off. The filtrated after removal of solvent was subjected to column chromatography packed with silica and ethyl acetate/petroleum ether was used as the eluant. Colourless slabs of (I) were grown by solvent evaporation from a solution of the compound in chloroform.

### Refinement

The H atoms of the water molecule were located in difference map and its positional parameters were refined freely [O3—H1W = 0.85 (5) and O3—H2W = 0.83 (4) Å]. The remaining H atoms were positioned geometrically, with O—H = 0.82 Å (for OH) and C—H = 0.93, 0.97 and 0.96 Å for aromatic, methylene and methyl H, respectively, and refined as riding with *U*_iso_(H) = 1.2 or 1.5 *U*_eq_(O, C).

### Crystal data

**Table:**

C_11_H_10_ClNO_2_·H_2_O	*F*(000) = 504
*M**_r_* = 241.67	*D*_x_ = 1.456 Mg m^−^^3^
Monoclinic, *P*2_1_/*n*	Mo *K*α radiation, λ = 0.71073 Å
Hall symbol: -P 2yn	Cell parameters from 1021 reflections
*a* = 9.161 (5) Å	θ = 1.9–20.2°
*b* = 14.246 (5) Å	µ = 0.34 mm^−^^1^
*c* = 9.464 (5) Å	*T* = 290 K
β = 116.819 (5)°	Slab, colourless
*V* = 1102.3 (9) Å^3^	0.31 × 0.21 × 0.10 mm
*Z* = 4	

### Data collection

**Table:**

Oxford Xcalibur Eos (Nova) CCD detector diffractometer	2044 independent reflections
Radiation source: Enhance (Mo) X-ray Source	1212 reflections with *I* > 2σ(*I*)
graphite	*R*_int_ = 0.100
ω scans	θ_max_ = 25.5°, θ_min_ = 2.9°
Absorption correction: multi-scan (*CrysAlis RED*; Oxford Diffraction, 2009)	*h* = −11→11
*T*_min_ = 0.903, *T*_max_ = 0.967	*k* = −17→17
8360 measured reflections	*l* = −11→11

### Refinement

**Table:**

Refinement on *F*^2^	Primary atom site location: structure-invariant direct methods
Least-squares matrix: full	Secondary atom site location: difference Fourier map
*R*[*F*^2^ > 2σ(*F*^2^)] = 0.054	Hydrogen site location: inferred from neighbouring sites
*wR*(*F*^2^) = 0.134	H atoms treated by a mixture of independent and constrained refinement
*S* = 0.90	*w* = 1/[σ^2^(*F*_o_^2^) + (0.0692*P*)^2^] where *P* = (*F*_o_^2^ + 2*F*_c_^2^)/3
2044 reflections	(Δ/σ)_max_ < 0.001
153 parameters	Δρ_max_ = 0.40 e Å^−^^3^
0 restraints	Δρ_min_ = −0.26 e Å^−^^3^

### Special details

**Table:**

Geometry. Bond distances, angles *etc*. have been calculated using the rounded fractional coordinates. All su's are estimated from the variances of the (full) variance-covariance matrix. The cell e.s.d.'s are taken into account in the estimation of distances, angles and torsion angles
Refinement. Refinement on *F*^2^ for ALL reflections except those flagged by the user for potential systematic errors. Weighted *R*-factors *wR* and all goodnesses of fit *S* are based on *F*^2^, conventional *R*-factors *R* are based on *F*, with *F* set to zero for negative *F*^2^. The observed criterion of *F*^2^ > σ(*F*^2^) is used only for calculating -*R*-factor-obs *etc*. and is not relevant to the choice of reflections for refinement. *R*-factors based on *F*^2^ are statistically about twice as large as those based on *F*, and *R*-factors based on ALL data will be even larger.

### Fractional atomic coordinates and isotropic or equivalent isotropic displacement parameters (Å^2^)

**Table:**

	*x*	*y*	*z*	*U*_iso_*/*U*_eq_	
Cl1	−0.12994 (10)	0.21700 (5)	−0.05478 (9)	0.0525 (3)	
O1	0.0858 (3)	0.2656 (2)	−0.3822 (3)	0.0713 (10)	
O2	0.1487 (3)	0.46372 (14)	0.3536 (2)	0.0515 (8)	
N1	0.0521 (3)	0.35801 (15)	0.0948 (3)	0.0371 (8)	
C1	0.0114 (4)	0.30580 (19)	−0.0297 (3)	0.0399 (10)	
C2	0.0664 (4)	0.3140 (2)	−0.1453 (3)	0.0435 (10)	
C3	0.1715 (4)	0.3870 (2)	−0.1243 (3)	0.0493 (11)	
C4	0.3261 (4)	0.5254 (2)	0.0313 (4)	0.0558 (12)	
C5	0.3720 (4)	0.5792 (2)	0.1620 (5)	0.0603 (14)	
C6	0.3158 (4)	0.5601 (2)	0.2743 (4)	0.0545 (12)	
C7	0.2105 (4)	0.4875 (2)	0.2525 (3)	0.0436 (10)	
C8	0.1595 (3)	0.43029 (18)	0.1160 (3)	0.0379 (9)	
C9	0.2198 (4)	0.4484 (2)	0.0056 (3)	0.0444 (11)	
C10	0.0147 (4)	0.2459 (3)	−0.2813 (4)	0.0573 (11)	
C11	0.2035 (5)	0.5135 (3)	0.4973 (4)	0.0678 (16)	
O3	0.8868 (3)	0.3074 (2)	0.2937 (3)	0.0716 (10)	
H1O	0.18120	0.24780	−0.34070	0.1070*	
H3	0.21180	0.39630	−0.19750	0.0590*	
H4	0.36470	0.53900	−0.04200	0.0670*	
H5	0.44190	0.62970	0.17760	0.0730*	
H6	0.35070	0.59720	0.36460	0.0660*	
H10A	0.04510	0.18290	−0.23950	0.0690*	
H10B	−0.10340	0.24780	−0.34210	0.0690*	
H11A	0.32050	0.50900	0.55460	0.1020*	
H11B	0.15520	0.48700	0.55960	0.1020*	
H11C	0.17230	0.57820	0.47550	0.1020*	
H1W	0.943 (5)	0.331 (3)	0.252 (5)	0.1080*	
H2W	0.949 (6)	0.301 (3)	0.389 (5)	0.1080*	

### Atomic displacement parameters (Å^2^)

**Table:**

	*U*^11^	*U*^22^	*U*^33^	*U*^12^	*U*^13^	*U*^23^
Cl1	0.0537 (5)	0.0487 (5)	0.0489 (5)	−0.0099 (4)	0.0176 (4)	−0.0054 (4)
O1	0.0644 (17)	0.112 (2)	0.0369 (13)	0.0262 (15)	0.0223 (13)	0.0030 (12)
O2	0.0555 (15)	0.0550 (14)	0.0428 (12)	−0.0026 (11)	0.0212 (11)	−0.0056 (10)
N1	0.0369 (14)	0.0372 (14)	0.0321 (13)	0.0029 (11)	0.0112 (11)	0.0046 (11)
C1	0.0379 (17)	0.0400 (17)	0.0342 (17)	0.0072 (14)	0.0096 (14)	0.0066 (13)
C2	0.0369 (17)	0.054 (2)	0.0324 (17)	0.0087 (15)	0.0094 (14)	0.0063 (13)
C3	0.047 (2)	0.067 (2)	0.0379 (18)	0.0167 (18)	0.0228 (16)	0.0161 (16)
C4	0.042 (2)	0.056 (2)	0.072 (2)	0.0051 (17)	0.0280 (18)	0.0169 (19)
C5	0.043 (2)	0.040 (2)	0.090 (3)	−0.0063 (16)	0.023 (2)	0.0051 (19)
C6	0.047 (2)	0.044 (2)	0.061 (2)	−0.0025 (16)	0.0141 (18)	−0.0045 (16)
C7	0.0358 (17)	0.0434 (18)	0.0448 (19)	0.0081 (15)	0.0122 (15)	0.0033 (14)
C8	0.0317 (16)	0.0343 (16)	0.0396 (16)	0.0055 (14)	0.0089 (14)	0.0054 (13)
C9	0.0380 (18)	0.0480 (19)	0.0458 (19)	0.0053 (15)	0.0176 (16)	0.0110 (15)
C10	0.056 (2)	0.076 (2)	0.0358 (19)	0.0121 (18)	0.0170 (17)	−0.0025 (16)
C11	0.071 (3)	0.078 (3)	0.046 (2)	0.000 (2)	0.019 (2)	−0.0121 (17)
O3	0.0575 (17)	0.108 (2)	0.0480 (15)	−0.0196 (15)	0.0226 (13)	0.0007 (15)

### Geometric parameters (Å, °)

**Table:**

Cl1—C1	1.748 (4)	C4—C9	1.413 (5)
O1—C10	1.406 (5)	C5—C6	1.401 (6)
O2—C7	1.356 (4)	C6—C7	1.365 (5)
O2—C11	1.410 (4)	C7—C8	1.417 (4)
O1—H1O	0.8200	C8—C9	1.409 (4)
O3—H1W	0.85 (5)	C3—H3	0.9300
O3—H2W	0.83 (4)	C4—H4	0.9300
N1—C1	1.298 (4)	C5—H5	0.9300
N1—C8	1.375 (4)	C6—H6	0.9300
C1—C2	1.401 (5)	C10—H10B	0.9700
C2—C3	1.369 (5)	C10—H10A	0.9700
C2—C10	1.507 (5)	C11—H11C	0.9600
C3—C9	1.408 (4)	C11—H11A	0.9600
C4—C5	1.351 (5)	C11—H11B	0.9600
			
Cl1···O2^i^	3.147 (3)		
			
C7—O2—C11	118.3 (3)	C3—C9—C8	117.4 (3)
C10—O1—H1O	109.00	O1—C10—C2	112.8 (3)
H1W—O3—H2W	107 (5)	C2—C3—H3	119.00
C1—N1—C8	117.1 (3)	C9—C3—H3	119.00
Cl1—C1—N1	115.5 (3)	C5—C4—H4	120.00
Cl1—C1—C2	117.4 (2)	C9—C4—H4	120.00
N1—C1—C2	127.2 (3)	C6—C5—H5	120.00
C1—C2—C10	121.9 (3)	C4—C5—H5	119.00
C1—C2—C3	115.3 (3)	C7—C6—H6	120.00
C3—C2—C10	122.8 (3)	C5—C6—H6	120.00
C2—C3—C9	121.5 (3)	O1—C10—H10A	109.00
C5—C4—C9	120.2 (3)	C2—C10—H10A	109.00
C4—C5—C6	121.0 (3)	C2—C10—H10B	109.00
C5—C6—C7	120.6 (3)	O1—C10—H10B	109.00
O2—C7—C8	115.4 (3)	H10A—C10—H10B	108.00
C6—C7—C8	119.6 (3)	O2—C11—H11B	109.00
O2—C7—C6	125.0 (3)	O2—C11—H11C	110.00
N1—C8—C9	121.6 (2)	O2—C11—H11A	109.00
N1—C8—C7	118.9 (3)	H11A—C11—H11C	109.00
C7—C8—C9	119.5 (3)	H11B—C11—H11C	110.00
C3—C9—C4	123.6 (3)	H11A—C11—H11B	109.00
C4—C9—C8	119.0 (3)		
			
C11—O2—C7—C6	−4.0 (5)	C2—C3—C9—C8	1.8 (5)
C11—O2—C7—C8	175.5 (3)	C9—C4—C5—C6	0.0 (6)
C8—N1—C1—Cl1	−178.2 (2)	C5—C4—C9—C3	−177.5 (3)
C8—N1—C1—C2	0.8 (5)	C5—C4—C9—C8	1.6 (5)
C1—N1—C8—C7	−178.4 (3)	C4—C5—C6—C7	−1.3 (6)
C1—N1—C8—C9	1.5 (4)	C5—C6—C7—O2	−179.7 (3)
Cl1—C1—C2—C3	177.3 (2)	C5—C6—C7—C8	0.9 (5)
Cl1—C1—C2—C10	−3.6 (4)	O2—C7—C8—N1	1.1 (4)
N1—C1—C2—C3	−1.7 (5)	O2—C7—C8—C9	−178.8 (3)
N1—C1—C2—C10	177.4 (3)	C6—C7—C8—N1	−179.3 (3)
C1—C2—C3—C9	0.3 (5)	C6—C7—C8—C9	0.8 (5)
C10—C2—C3—C9	−178.8 (3)	N1—C8—C9—C3	−2.7 (4)
C1—C2—C10—O1	−179.0 (3)	N1—C8—C9—C4	178.1 (3)
C3—C2—C10—O1	0.1 (5)	C7—C8—C9—C3	177.1 (3)
C2—C3—C9—C4	−179.1 (3)	C7—C8—C9—C4	−2.0 (4)

Symmetry codes: (i) *x*−1/2, −*y*+1/2, *z*−1/2.

### Hydrogen-bond geometry (Å, °)

**Table:**

Cg1 is the centroid of the N1/C1–C3/C8/C9 ring.

**Table:**

*D*—H···*A*	*D*—H	H···*A*	*D*···*A*	*D*—H···*A*
O1—H1O···O3^i^	0.82	1.90	2.705 (4)	165
O3—H1W···N1^ii^	0.85 (5)	2.17 (5)	2.988 (4)	163 (4)
O3—H2W···O1^iii^	0.83 (4)	2.02 (4)	2.836 (4)	171 (5)
C10—H10B···Cg1^i^	0.97	2.93	3.738 (5)	142

Symmetry codes: (i) *x*−1/2, −*y*+1/2, *z*−1/2; (ii) *x*+1, *y*, *z*; (iii) *x*+1, *y*, *z*+1.
